# Bad Feet

**Published:** 1875-12

**Authors:** 


### BAD FEET.

Canova chose five hundred of most beautiful women from whom to model his Venus, and among them all could not find a decent set of toes. If he had lived nowadays what luck would he have had under the dainty little buttoned boots, with their sharp-pointed heels? If adult women, however, choose to torture themselves, there is no reason why any one should remonstrate; but the condition of the feet of the children is becoming too serious a matter to be passed by in silence. As soon as the helpless baby can put its foot to the ground, and before it can complain in words, shoes are put on it by which the width of the toes is contracted fully half an inch; and usually a stiff counter is ordered in the heel with some vague idea of "strengthening the ankle." From that time, no matter how watchful or sensible its parents may be in other respects, these instruments of torture always constitute part of its dress. The toes are forced into a narrower space year by year, "to give a good shape to the foot," until they overlap and knot and knob themselves over with incipient corns and bunions. Then the heel is lifted from the ground by artificial means, and thus the action of the calf muscles is hindered and the elastic cartilage of the whole foot is stiffened at the earliest and most tender period of its growth. The results are a total lack of elasticity in the step and carriage and a foot inevitably distorted and diseased. American women are noted for their cramped and mincing walk. Southern children are more fortunate in this matter than those in the North, as it is customary, even in the wealthiest clases, to let their feet go uncovered until the age of six. Mothers in the North are not wholly to blame, however, as the climate requires that the feet shall be covered, and it is almost impossible, even in New York, to find shoes properly made for children, unless a last is ordered for the foot. As a new last would be required every month or two, very few parents are able to give the watchfulness and money required; but if the proper shape were insisted upon by those buying shoes, dealers would quickly furnish them. Nothing is more prompt than the reply of trade to any hint of a new want or fashion. McComber's last is the best we have ever seen.

